# The Efficacy and Safety of Compound Danshen Dripping Pill Combined with Percutaneous Coronary Intervention for Coronary Heart Disease

**DOI:** 10.1155/2020/5067137

**Published:** 2020-11-17

**Authors:** Cailan Li, Qian Li, Jiamin Xu, Wenzhen Wu, Yuling Wu, Jianhui Xie, Xiaobo Yang

**Affiliations:** ^1^Department of Pharmacology, Zunyi Medical University, Zhuhai Campus, Zhuhai 519041, China; ^2^State Key Laboratory of Dampness Syndrome of Chinese Medicine, The Second Affiliated Hospital of Guangzhou University of Chinese Medicine, Guangzhou 510120, China; ^3^Guangdong Provincial Key Laboratory of Clinical Research on Traditional Chinese Medicine Syndrome, The Second Affiliated Hospital of Guangzhou University of Chinese Medicine, Guangzhou 510120, China; ^4^The Second School of Medicine, Guangzhou University of Chinese Medicine, Guangzhou 510120, China

## Abstract

**Objective:**

Compound Danshen dripping pill (CDDP) is a well-known Chinese patent medicine, which is commonly used for the treatment of coronary heart disease (CHD) in China. This study is aimed at systematically assessing the clinical efficacy of CDDP for CHD patients.

**Methods:**

Eight databases were retrieved for eligible research studies from the founding date to April 20, 2020. Risk ratio (RR) was used to assess major adverse cardiac events (MACE) and adverse reactions, and mean difference (MD) was adopted to evaluate the hemorheology and blood lipid indexes, vascular endothelial function, cardiac function, and inflammation.

**Result:**

Twenty randomized controlled trials involving 2574 participants with CHD were included. The results indicated that, compared with percutaneous coronary intervention (PCI) alone, the combination of CDDP with PCI treatment remarkably reduced MACE (RR = 0.53, 95% confidence interval (CI) (0.44, 0.65), *P* < 0.00001). Moreover, hemorheology and blood lipid parameters and inflammatory mediators of CHD patients were also dramatically mitigated after the combined therapy (*P* < 0.01). In addition, vascular endothelial function and cardiac function were prominently improved by this combination (*P* < 0.001). However, there was no significant difference in adverse reactions between the two groups (*P* > 0.05).

**Conclusion:**

Evidence from the meta-analysis demonstrated that CDDP combined with PCI treatment prominently reduced the incidence of MACE, improved cardiovascular functions, and inhibited inflammation in CHD patients. Therefore, CDDP combined with PCI treatment could be an effective and safe therapeutic method for CHD patients.

## 1. Introduction

Coronary heart disease (CHD) is one of the most serious heart diseases that threaten human health [1]. It is characterized by coronary atherosclerosis lesions aroused by myocardial ischemia, hypoxia or necrosis, stenosis, occlusion of the lumen, and inflammation [2]. The World Health Organization (WHO) divides CHD into five categories: asymptomatic myocardial ischemia, angina pectoris, myocardial infarction, ischemic cardiomyopathy, and sudden death [3]. In recent years, in order to keep up with the continuous updating of the concept of diagnosis and treatment of CHD and facilitate the formulation of treatment strategies, two kinds of syndromes were proposed clinically, namely, chronic myocardial ischemia syndrome and acute coronary syndrome (ACS) [4, 5]. CHD is one of the major causes of death worldwide, accounting for about one-third of all deaths [6].

The treatment of CHD mainly includes lifestyle change, drug therapy, percutaneous coronary intervention (PCI), and surgical operation [7]. At present, PCI has been the most common method for the treatment of CHD, which can effectively alleviate coronary artery stenosis or occlusion, rebuild coronary artery blood flow, and improve coronary artery blood circulation, with less trauma and obvious clinical effect [8]. However, PCI is an invasive operation, which very easily leads to vascular endothelial injury [9]. After PCI treatment, there was a high incidence of adverse cardiovascular events such as recurrent angina pectoris, coronary restenosis, acute myocardial infarction, malignant arrhythmia, and sudden death, which reduces the therapeutic effect of PCI treatment for CHD patients [10]. Therefore, how to improve the short-term efficacy and long-term prognosis of CHD patients with PCI treatment has become the direction clinical workers should strive for.

According to the basic theory of traditional Chinese medicine, the etiology and pathogenesis of CHD are associated with blood stasis. Therefore, promoting blood circulation and removing blood stasis are an important treatment for CHD. Compound Danshen dripping pill (CDDP) is a famous Chinese patent medicine approved by China Food and Drug Administration (CFDA), which has been widely used in various cardiocerebrovascular diseases [11, 12]. CDDP is prepared from *Salviae miltiorrhizae*, *Panax notoginseng*, and *Borneolum* with modern techniques [13]. The main function of CDDP is promoting blood circulation to remove blood stasis and regulating qi-flowing for relieving pain [14]. In recent years, CDDP is frequently used to treat CHD sufferers combined with PCI [15]. However, most of the clinical researches could not provide sufficient evidence for the small sample sizes, and systematic evidence is lacking and urgently needed to prove the efficacy and safety. Therefore, this meta-analysis was conducted by systematically evaluating the effectiveness of CDDP combined with PCI for CHD compared with PCI therapy alone, in order to provide a scientific basis for this combination treatment.

## 2. Materials and Methods

### 2.1. Search Strategy

This meta-analysis was conducted according to the PRISMA statement [16]. Randomized controlled trials (RCTs) were independently searched and retrieved by two investigators (Cailan Li and Qian Li) in the following databases from the founding date to April 20, 2020: PubMed, Embase, the Cochrane Library, Web of Science (WOS), China National Knowledge Infrastructure (CNKI), China Biology Medicine disc (CBMdisc), Wanfang data, and VIP medicine information system (VMIS). In the literature retrieval, the following search terms were used in combination: (“compound Danshen dripping pill” OR “Fufang Danshen diwan”) AND (“coronary heart disease” OR “CHD” OR “coronary artery disease” OR “CAD”) AND (“percutaneous coronary intervention” OR “PCI”).

### 2.2. Inclusion Criteria

Based on the suggestions of several specialists, the inclusion criteria were established as follows: (1) participants were diagnosed with CHD by the cardiovascular disease diagnostic criteria determined by the Chinese Medical Society (CMA) or American Heart Association (AHA) in RCTs [17, 18]; (2) all researches mentioned were described as RCTs; (3) CDDP served as the only Chinese patent medicine in RCTs; (4) sufferers in the experimental group were treated with the combined therapy of CDDP and PCI-based treatment, whereas sufferers in the control group only received PCI therapy; (5) outcome measurements of each research included at least one of the following indices: major adverse cardiac events (MACE) including recurrent angina, coronary restenosis, acute myocardial infarction, malignant arrhythmia, cardiac failure, cardiogenic shock and sudden cardiac death, hemorheology indices including whole blood viscosity (WBV), plasma viscosity (PV), hematocrit (HCT), erythrocyte aggregation index (EAI), and fibrinogen (FIB) level, vascular endothelial function indicators involving endothelin (ET), flow mediated dilation (FMD), and nitric oxide (NO), blood lipid parameters including total cholesterol (TC), triglyceride (TG), high density lipoprotein cholesterol (HDL-C), and low density lipoprotein cholesterol (LDL-C), cardiac function indicators including left ventricular ejection fraction (LVEF), left ventricular end diastolic diameter (LVEDD), and cardiac index (CI), inflammatory mediators including high-sensitivity C-reactive protein (Hs-CRP), tumor necrosis factor-alpha (TNF-*α*), interleukin-6 (IL-6) and interleukin-8 (IL-8), and adverse reactions. Among these indices, MACE is the primary indicator, and the others belong to the secondary indicators.

### 2.3. Exclusion Criteria

If they demonstrated any one of the following, researches could be ruled out: (1) they were case report, editorials, and irrelevant clinical trials; (2) studies were not RCTs or diagnostic criteria were not clear; (3) the intervention of CHD patients was not conformed; (4) for the researches with data duplication, the late published study was regarded as data fraud and rejected if the authors could not be reached.

### 2.4. Data Extraction and Quality Evaluation

Information of eligible researches containing author names, publication year, sample size, intervention methods, outcome measurements, etc. was abstracted and is summarized in Table 1. In light of the Cochrane Handbook for Systematic Reviews of Interventions, quality assessment of included studies was independently conducted by two authors (Cailan Li and Jiamin Xu) using the risk of bias table from Review Manager 5.3 [39]. There were seven kinds of biases including random sequence generation (selection bias), allocation concealment (selection bias), blinding of participants and personnel (performance bias), blinding of outcome assessment (detection bias), incomplete outcome data (attrition bias), selective reporting (reporting bias), and other bias. Each term was judged with three grades: low risk of bias, unclear, and high risk of bias. “Low risk of bias” indicates the description of methods or procedures was adequate or correct, while “high risk of bias” represents inadequate or incorrect description. When inadequate information was shown in the study and we could not definitely judge “high risk” or “low risk,” the item was judged as “unclear.” Discrepancies about data abstraction and research assessment were settled by mutual discussion or consultation to a third reviewer (Jianhui Xie).

### 2.5. Statistical Analysis

Review Manager 5.3 (Cochrane Collaboration) was used to analyze the abstracted data from the eligible researches [39]. Outcome measures including MACE and adverse reactions were considered as dichotomous variables and presented as the risk ratio (RR) with 95% confidence interval (CI), the indices of hemorheology, vascular endothelial function, blood lipid, cardiac function, and inflammation being continuous variables that presented as the mean difference (MD) with 95% CI. The chi-squared test was employed to check the heterogeneity among researches, and *I*^2^ statistic was used for showing the size of heterogeneity. A fixed-effect model was adopted to analyze data with low heterogeneity (*P* ≥ 0.1 and *I*^2^ ≤ 50%), and data with high heterogeneity (*P* < 0.1 or *I*^2^ > 50%) was evaluated by a random-effect model [40]. The risk of publication bias in the included researches was revealed by a funnel plot.

## 3. Results

### 3.1. Study Selection

**Figure 1:**
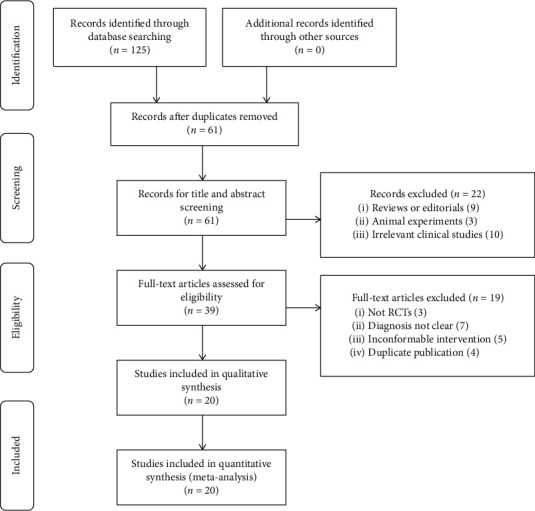
Flow diagram of study searching and screening for the meta-analysis.

There were one hundred and twenty-five potential records from Chinese databases identified in the first review, and no related record was retrieved in English databases. Sixty-four duplicated records were removed due to the intersection of database coverage. A total of 61 records were obtained for title or abstract examination, and 22 records were dropped by reason of unrelated topics. Thirty-nine records were reserved to check full text. According to the full-text inspection, 19 studies were excluded for the following reasons: 3 researches were not RCTs, diagnosis in 7 studies was not clear, 5 trials mentioned improper interventions, and 4 articles,, respectively, showed same data with another article. In final, there were twenty researches [19–38] included in the meta-analysis (Figure 1).

### 3.2. Study Characteristics and Quality Assessment

Twenty eligible researches including 2574 sufferers were all published in Chinese databases from 2003 to 2019. The experimental group contained 1281 sufferers, and the control group contained 1293 sufferers. The age of all the sufferers ranged from 20 to 80. All the included trials were RCTs with a comparison between the combined therapy of CDDP and PCI treatment and PCI treatment only. In all studies, the dose of CDDP was 10 pills each time, three times a day by oral administration. And most studies reported that the duration of treatment lasted for 3 months or so. Obvious difference was not found between the two groups in basic information (Table 1).

**Figure 2:**
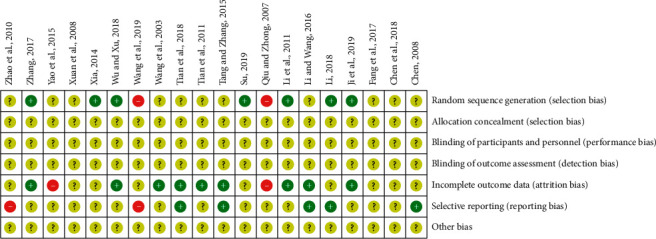
Methodological quality assessment for the risk of bias in the included studies.

The methodological quality of the eligible studies was assessed by the Cochrane risk of bias evaluation and showed to be universally low. Eleven [19, 21–23, 25, 27–30, 32, 34] of the included trials showed the allocation sequence generation without giving the specific random method, and seven studies [24, 26, 31, 33, 35–37] indicated that they were randomly grouped according to the random number table method. All the included researches did not describe allocation concealment, blinding of participants, and outcome assessment. Nine trials [19, 24, 25, 27, 29, 31, 34–36] were at low risk of attrition bias for providing a complete outcome data. Five researches [21, 27, 29, 33, 34] reporting the result of detailed indices showed a low risk of reporting bias. The risk of bias graph is shown in Figure 2.

### 3.3. Major Adverse cardiac events

**Figure 3:**
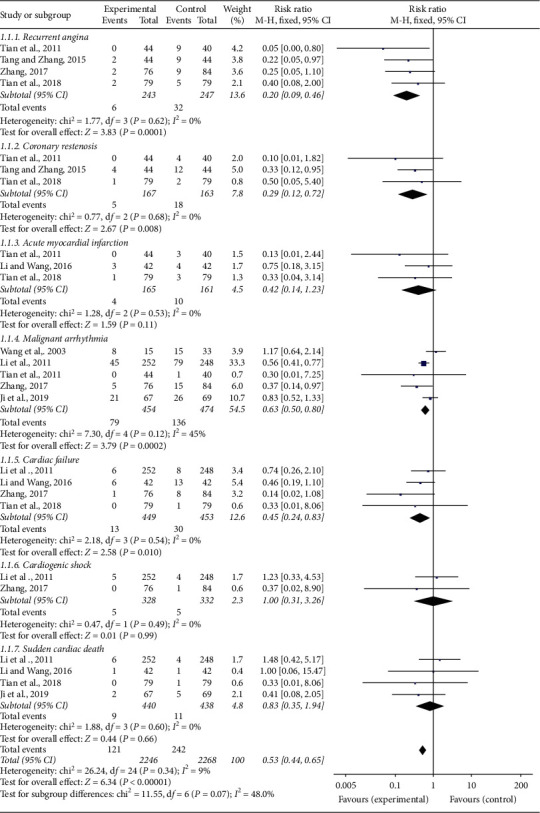
Forest plot of major adverse cardiac events of CDDP plus PCI treatment compared to PCI treatment alone for CHD patients. *I*^2^ and *P* are the criterion for the heterogeneity test, ◆ pooled risk ratio, –■– risk ratio, and 95% CI.

Eight [19, 24, 25, 27, 29, 31, 34, 36] of 20 researches compared the incidence of MACE between CDDP combined with PCI treatment and single PCI treatment. A meta-analysis of the 8 studies adopting a fixed-effect model indicated that the combination therapy of CDDP and PCI treatment markedly reduced the incidence of MACE compared to single PCI treatment in treating CHD (RR = 0.53, 95% CI (0.44, 0.65), *P* < 0.00001). No statistically significant heterogeneity (*P* = 0.34, *I*^2^ = 9%) was found among individual studies (Figure 3).

Furthermore, subgroup analysis was performed based on different cardiac events. There were, respectively, four [25, 27, 31, 34], three [25, 27, 34], three [25, 29, 34], five [19, 24, 25, 31, 36], four [24, 29, 31, 34], two [24, 31], and four [24, 29, 34, 36] trials comparing the incidence of recurrent angina, coronary restenosis, acute myocardial infarction, malignant arrhythmia, cardiac failure, cardiogenic shock, and sudden cardiac death between the experimental and control groups. The results of subgroup analysis showed that CDDP could significantly reduce the incidence of recurrent angina (RR = 0.20, 95% CI (0.09, 0.46), *P* = 0.0001), coronary restenosis (RR = 0.29, 95% CI (0.12, 0.72), *P* = 0.008), malignant arrhythmia (RR = 0.63, 95% CI (0.50, 0.80), *P* = 0.0002), and cardiac failure (RR = 0.45, 95% CI (0.24, 0.83), *P* = 0.01), and there was no difference about the incidence of acute myocardial infarction (RR = 0.42, 95% CI (0.14, 1.23), *P* = 0.11), cardiogenic shock (RR = 1, 95% CI (0.31, 3.26), *P* = 0.99), and sudden cardiac death (RR = 0.83, 95% CI (0.35, 1.94), *P* = 0.66) between the experimental and control groups (Figure 3).

### 3.4. Hemorheology Indices

**Figure 4:**
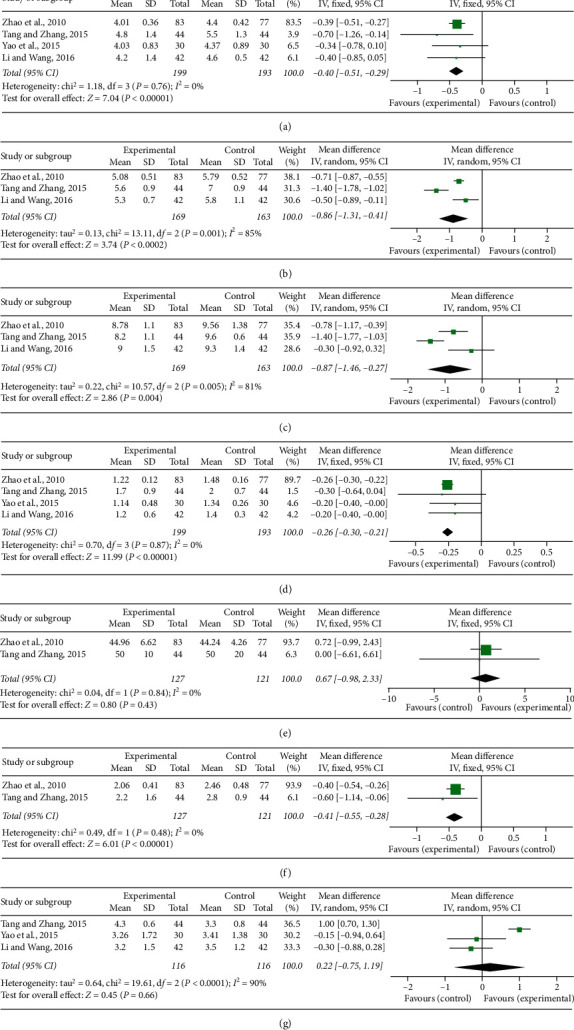
Forest plot of comparison in two groups for hemorheology indices.(a)-(c) Whole blood viscosity (high, middle, and low shear); (d) plasma viscosity; (e) hematocrit; (f) erythrocyte aggregation index; (g) fibrinogen level. *I*^2^ and *P* are the criterion for the heterogeneity test, ◆ pooled mean difference, –■– mean difference, and 95% CI.

WBV, PV, HCT, EAI, and FIB were the indices of blood rheology measured in the eligible researches. There were four trials [23, 27–29] mentioned WBV (high shear). No heterogeneity was found among individual researches (*P* = 0.76, *I*^2^ = 0%), so a fixed-effect model was used to conduct a meta-analysis which showed that CDDP combined with PCI treatment markedly reduced WBV (high shear) (MD = −0.4, 95% CI (−0.51, −0.29), *P* < 0.00001; Figure 4(a). Three trials [23, 27, 29] compared WBV (middle and low shear) between the experimental and control groups. Significant heterogeneity was, respectively, found among individual researches (*P* = 0.001, *I*^2^ = 85%; *P* = 0.005, *I*^2^ = 81%), and a random-effect model was adopted to carry out the meta-analysis. The pooled results showed that the combination of CDDP and PCI treatment significantly decreased WBV (middle shear) (MD = −0.86, 95% CI (−1.31, −0.41), *P* = 0.0002; Figure 4(b)) and WBV (low shear) (MD = -0.87, 95% CI (−1.46, −0.27), *P* = 0.0004; Figure 4(c)).

There were, respectively, four studies [23, 27–29] that reported PV and two studies [23, 27] that reported HCT  and EAI. No heterogeneity was, respectively, found among individual researches (*P* = 0.87, *I*^2^ = 0%; *P* = 0.84, *I*^2^ = 0%; *P* = 0.48, *I*^2^ = 0%), and a fixed-effect model was adopted to carry out the meta-analysis. The pooled results showed that the combination of CDDP and PCI treatment significantly decreased PV (MD = −0.26, 95% CI (−0.3, −0.21), *P* < 0.00001; Figure 4(d)) and EAI (MD = −0.41, 95% CI (−0.55, −0.28), *P* < 0.00001; Figure 4(f)), and there was no difference about HCT between the experimental and control groups (MD = 0.67, 95% CI (−0.98, 2.33), *P* = 0.43; Figure 4(e)).

Three studies [23, 27, 29] reported the level of FIB in blood plasma. Significant heterogeneity was observed among individual researches (*P* < 0.0001, *I*^2^ = 90%) and then a random-effect meta-analysis was conducted to indicate that there was no difference about FIB between the experimental and control groups (MD = 0.22, 95% CI (−0.75, 1.19), *P* = 0.66; Figure 4(g)).

### 3.5. Vascular Endothelial Function Indices

**Figure 5:**
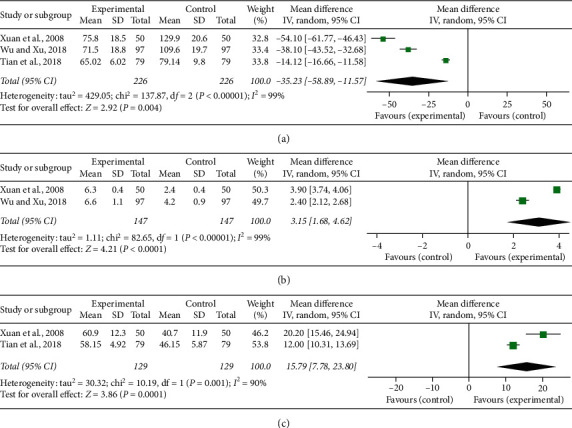
Forest plot of comparison in two groups for vascular endothelial function indices. (a) Endothelin; (b) flow mediated dilation; (c) nitric oxide. *I*^2^ and *P* are the criterion for the heterogeneity test, ◆ pooled mean difference, –■– mean difference, and 95% CI.

ET, FMD, and NO were the indices of vascular endothelial function measured in the included studies. There were, respectively, three [22, 34, 35], two [22, 35], and two [22, 34] studies reporting ET, FMD, and NO. Significant heterogeneity was, respectively, found among individual studies (*P* < 0.00001, *I*^2^ = 99%; *P* < 0.00001, *I*^2^ = 99%; *P* = 0.001, *I*^2^ = 90%), and a random-effect model was adopted to carry out the meta-analysis. The pooled results showed that the combination of CDDP and PCI treatment significantly decreased ET (MD = −35.23, 95% CI (−58.89, −11.57), *P* = 0.004; Figure 5(a)) and improved FMD (MD = 3.15, 95% CI (1.68, 4.62), *P* < 0.0001; Figure 5(b)) and NO (MD = 15.79, 95% CI (7.78, 23.8), *P* = 0.0001; Figure 5(c)).

### 3.6. Blood Lipid Indices

**Figure 6:**
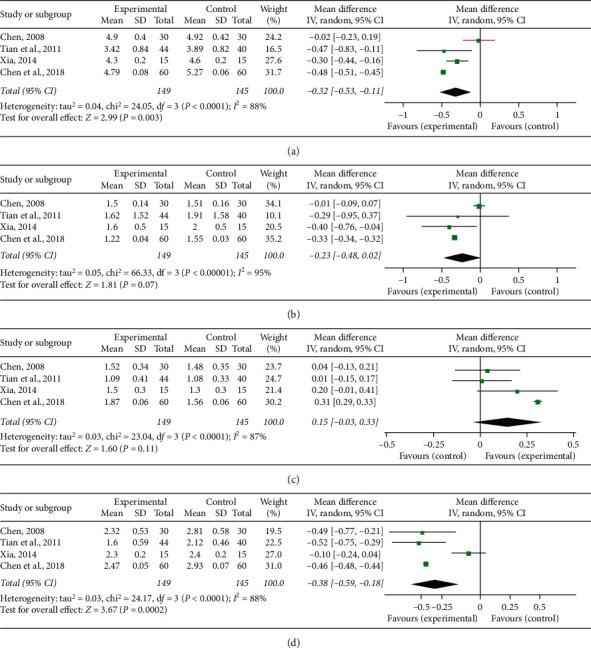
Forest plot of comparison in two groups for blood lipid indices. (a) Total cholesterol; (b) triglyceride; (c) high density lipoprotein cholesterol; (d) low density lipoprotein cholesterol. *I*^2^ and *P* are the criterion for the heterogeneity test, ◆ pooled mean difference, –■– mean difference, and 95% CI.

TC, TG, HDL-C, and LDL-C were the indices of blood lipid measured in the included studies. There were four studies [21, 25, 26, 32] that reported TC, TG, HDL-C, and LDL-C. Significant heterogeneity was, respectively, found among individual researches (*P* < 0.00001, *I*^2^ = 88%; *P* < 0.00001, *I*^2^ = 95%; *P* < 0.00001, *I*^2^ = 87%; *P* < 0.00001, *I*^2^ = 88%), and a random-effect model was adopted to carry out the meta-analysis. The pooled results showed that CDDP combined with PCI treatment significantly decreased TC (MD = −0.32, 95% CI (−0.53, −0.11), *P* = 0.003; Figure 6(a)) and LDL-C (MD = −0.38, 95% CI (−0.59, −0.18), *P* = 0.0002; Figure 6(d)), and there was no difference about TG (MD = −0.23, 95% CI (−0.48, 0.02), *P* = 0.07; Figure 6(b)) and HDL-C (MD = 0.15, 95% CI (−0.03, 0.33), *P* = 0.11; Figure 6(c)) between the experimental and control groups.

### 3.7. Cardiac Function Indices

**Figure 7:**
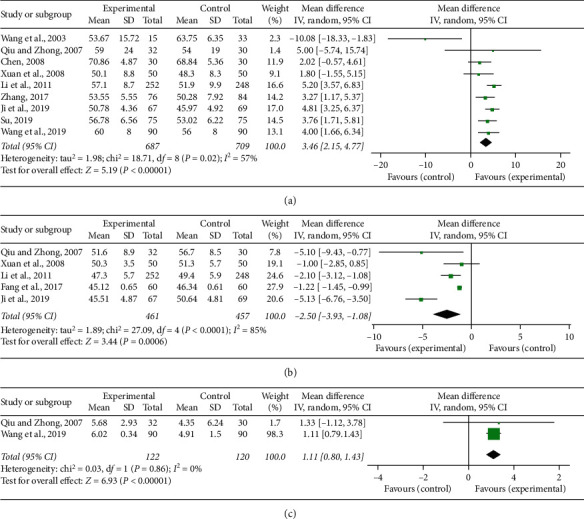
Forest plot of comparison in two groups for cardiac function indices. (a) Left ventricular ejection fraction; (b) left ventricular end diastolic diameter; (c) cardiac index. *I*^2^ and *P* are the criterion for the heterogeneity test, ◆ pooled mean difference, –■– mean difference, and 95% CI.

LVEF, LVEDD, and CI were the indices of cardiac function measured in the included studies. There were nine [19–22, 24, 31, 36–38] and five [20, 22, 24, 30, 36] studies which reported LVEF and LVEDD. Significant heterogeneity was, respectively, found among individual studies (*P* = 0.02, *I*^2^ = 57%; *P* < 0.0001, *I*^2^ = 85%), and a random-effect model was adopted to carry out the meta-analysis. The pooled results showed that CDDP combined with PCI treatment significantly improved LVEF (MD = 3.46, 95% CI (2.15, 4.77), *P* < 0.0001; Figure 7(a)) and decreased LVEDD (MD = -2.5, 95% CI (−3.93, −1.08), *P* = 0.0006; Figure 7(b)). Two studies [20, 38] recorded the detection of CI. There was no heterogeneity (*P* = 0.86, *I*^2^ = 0%) and a fixed-effect model was adopted to carry out the meta-analysis. The pooled result showed that the combination therapy of CDDP and PCI treatment significantly improved CI compared to single PCI treatment (MD = 1.11, 95% CI (0.8, 1.43), *P* < 0.00001; Figure 7(c)).

### 3.8. Inflammatory Mediators Production

**Figure 8:**
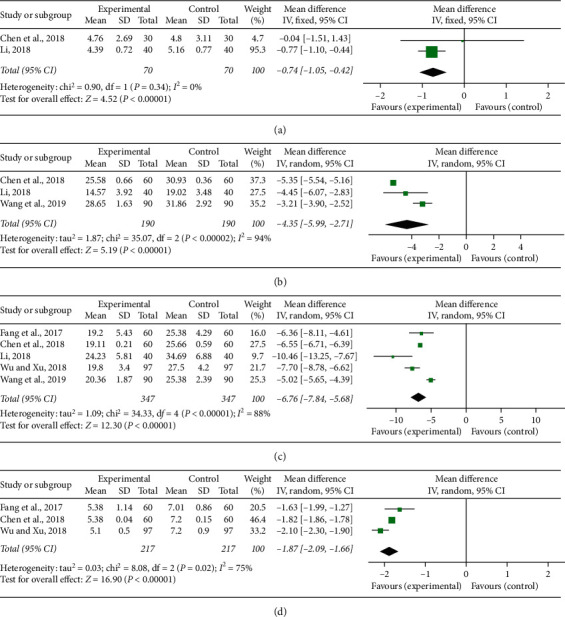
Forest plot of comparison in two groups for inflammatory indices. (a) High-sensitivity C-reactive protein; (b) tumor necrosis factor-alpha; (c) interleukin-6; (d) interleukin-8. *I*^2^ and *P* are the criterion for the heterogeneity test, ◆ pooled mean difference, –■– mean difference, and 95% CI.

Hs-CRP, TNF-*α*, IL-6, and IL-8 were the indices of inflammation measured in the included studies. Two studies [21, 33] mentioned the investigation on Hs-CRP. No statistically significant heterogeneity (*P* = 0.34, *I*^2^ = 0%) was detected in the meta-analysis and a fixed-effect model was used. An OR with 95% CI was adopted to present the comparison of Hs-CRP between the experimental and control groups (MD = −0.74, 95% CI (−1.05, −0.42), *P* < 0.00001; Figure 8(a)). It indicated that CDDP could significantly decrease Hs-CRP for CHD patients. There were three [32, 33, 38], five [30, 32, 33, 35, 38], and three [30, 32, 35] studies that reported TNF-*α*, IL-6, and IL-8. Significant heterogeneity was respectively, found, among individual researches (*P* < 0.00001, *I*^2^ = 94%; *P* < 0.00001, *I*^2^ = 88%; *P* = 0.02, *I*^2^ = 75%), and a random-effect model was adopted to carry out the meta-analysis. The pooled results showed that CDDP combined with PCI treatment significantly improved TNF-*α* (MD = −4.35, 95% CI (−5.99, −2.71), *P* < 0.00001; Figure 8(b)), IL-6 (MD = −6.76, 95% CI (−7.84, −5.68), *P* < 0.00001; Figure 8(c)), and IL-8 (MD = −1.87, 95% CI (−2.09, −1.66), *P* < 0.00001; Figure 8(d)).

### 3.9. Adverse Reaction

**Figure 9:**
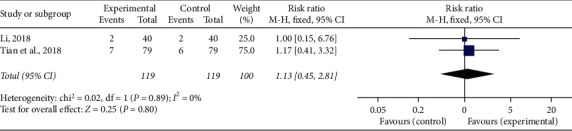
Forest plot of adverse reactions of CDDP plus PCI treatment compared to PCI treatment alone for CHD patients. *I*^2^ and *P* are the criterion for the heterogeneity test, ◆ pooled risk ratio, –■– risk ratio, and 95% CI.

Two [21, 28] of the included researches reported that no obvious adverse reaction occurred during treatment, and two [33, 34] recorded the incidence of adverse reactions. The adverse reactions consisted of gastrointestinal intolerance, dizziness, phlebitis, and pruritus. No heterogeneity (*P* = 0.89, *I*^2^ = 0%) was found among individual studies, and a fixed-effect model was used to perform the meta-analysis. The pooled RR with 95% CI showed that there was no difference about the incidence of adverse reactions between the experimental and control groups (RR = 1.13, 95% CI (0.45, 2.81), *P* = 0.8; Figure 9).

### 3.10. Publication Bias

**Figure 10:**
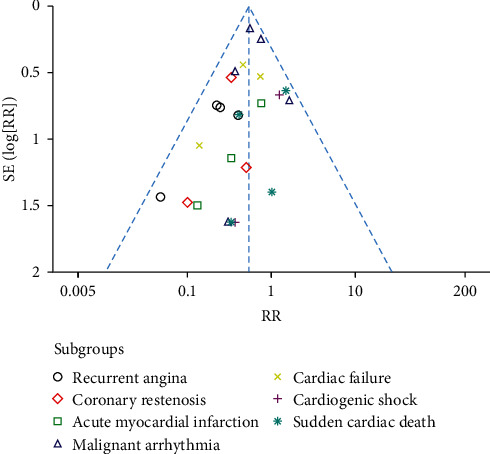
Funnel plot for the publication bias of major adverse cardiac events.

Funnel plot was employed to evaluate the publication bias. The publication bias was checked for MACE. As shown in Figure 10, the plots were basically symmetric, indicating that there was no obvious publication bias.

## 4. Discussion

### 4.1. Overview

Cardiovascular disease (CVD) is induced by more and more risk factors including improvement of people's living standards, changes in people's living habits, aging of population, and the constantly changing environment [41, 42]. The morbidity and mortality of CVD remain high, and the burden of prevention and treatment of CVD is increasing [43]. It has become an important public health issue for human health. CHD is one of the most common and harmful CVD, characterized by high disability rate, mortality rate, and many complications, and seriously threatens public health [44]. Therefore, the researches on the treatment of CHD are of great significance to human health. At present, PCI has become one of the main means for treating CHD because of its good therapeutic effect [45]. However, there has been a high incidence of adverse cardiovascular events in CHD patients after PCI treatment [46]. Given the circumstances, more effective and safe treatment is urgently needed for CHD patients in China and even the world.

Chinese clinicians have been looking for better treatments for CHD over the years. Traditional Chinese medicine (TCM) has been used to treat CHD for more than two thousand years. The therapeutic effect of Chinese traditional medicines for treating CHD is not bad and even stronger than some western medicines, and Chinese traditional medicines are characterized by little toxicity and side effect. Therefore, the application prospect of Chinese traditional medicines in CHD is great [47]. Along with the development of modern pharmaceutical technologies, oral preparations and injections for the prevention and treatment of CHD based on classical TCM prescriptions or theories have sprung up in large numbers [48].

CDDP is an excellent Chinese patent medicine developed by modern pharmaceutical technology based on the basic theory of TCM. Compared with the original tablet, CDDP has many advantages including small dosage, outstanding therapeutic effect, fewer side effects, and reduced gastrointestinal irritation [49]. Therefore, it is a commonly used Chinese medicine preparation in clinical practice in China. At present, Phase III clinical trial of CDDP has been completed in the United States. Then Tianjin Tasly Pharmaceutical Co., Ltd., will submit a new drug application to Food and Drug Administration [50]. CDDP is prepared from *Salviae miltiorrhizae*, *Panax notoginseng*, and *Borneolum*, and its major active constituents are tanshinol, protocatechuic aldehyde, salvianolic acid B, notoginsenoside, and so on [51]. Modern pharmacological studies have shown that these components are related to some effects, such as regulating lipid metabolism, improving vascular function, and inhibiting thrombosis [52]. CDDP have been widely used in the treatment of various CVD for many years [53]. However, there is lack of a comprehensive and systematic evaluation of CDDP for the treatment of CHD after PCI according to general international standards. Therefore, this study is aimed at providing an internationally recognized systematic assessment of the efficacy and safety of CDDP for treating CHD patients after PCI treatment.

This meta-analysis for the first time systematically assessed the clinical effect and safety of CDDP for treating CHD patients after PCI treatment. The incidence of MACE was used to evaluate the efficacy of CDDP combined with PCI treatment for CHD patients. Compared with PCI treatment alone, CDDP combined with PCI treatment was associated with remarkably lower MACE (*P* < 0.00001). Hemorheology indices, including WBV, PV, HCT, EAI, and FIB, were used to study the fluidity and deformability of blood in CHD participants. Compared with PCI treatment alone, CDDP combined with PCI treatment was associated with significantly lower WBV, PV, and EAI (*P* < 0.01). It indicated that CDDP contributed to improving the antithrombotic and anticoagulation effects. ET, FMD, and NO were used to evaluate vascular endothelial function in CHD patients. Our analysis results showed that, compared with PCI treatment alone, CDDP combined with PCI treatment was associated with significantly lower ET and higher FMD and NO (*P* < 0.01). Moreover, TC, TG, HDL-C, and LDL-C were used to assess the blood lipid in CHD patients. Results demonstrated that CDDP combined with PCI treatment significantly decreased the levels of TC and LDL-C in comparison with PCI treatment alone (*P* < 0.01). LVEF, LVEDD, and CI were used to estimate cardiac function in CHD patients. Compared with PCI treatment alone, CDDP combined with PCI treatment was associated with significantly lower LVEDD and higher LVEF and CI (*P* < 0.001). In addition, Hs-CRP, TNF-*α*, IL-6, and IL-8 were applied to evaluate inflammation state in CHD patients after PCI. Results suggested that, compared with PCI treatment alone, CDDP combined with PCI treatment was associated with significantly lower Hs-CRP, TNF-*α*, IL-6, and IL-8 (*P* < 0.00001). However, there was no difference about adverse reactions between the experimental and control groups (*P* = 0.8). It could be only temporarily concluded that CDDP is relatively safe without increasing the incidence of adverse reactions before including more eligible studies.

### 4.2. Limitations

Although comprehensive search and strict methodologies were employed to screen researches and investigate the therapeutic effect and safety associated with CDDP treatment, several potential limitations still existed in this meta-analysis that should be considered. Firstly, although an overall retrieval strategy was adopted to reduce the publication bias as far as possible, there was still a certain degree of selective bias that our meta-analysis only searched the Chinese and English databases and no reference was made to researches published in other languages. Secondly, all the eligible trials were conducted in China and most participants were Chinese. However, it is necessary to include some diverse population samples into the study to achieve more abundant and reliable results. Thirdly, the methodological quality in most of the eligible researches showed to be poor. Eleven of the 20 trials only referred to “randomization” but did not point out the specific random method. And all the included researches did not report allocation concealment and blinding method. Fourthly, we did not contact the authors using phone call or e-mail for more details of the included trials. Fifthly, there was statistically significant heterogeneity detected in several outcomes, such as FIB, ET, FMD, and NO. It is relatively difficult to study the heterogeneity in the outcomes of continuous variables. We were unable to perform a subgroup analysis for the small number of researches providing these outcomes and also failed to detect the sources of the heterogeneity after performing sensitivity analysis. It can be concluded that the heterogeneity came from two or more factors, such as gender, age, and duration of treatment. Finally, drug safety is significant to develop alternative medicines for health care. However, only two of the included researches reported adverse reactions.

### 4.3. Direction for the Future

According to our study, CDDP combined with PCI treatment is more effective for CHD patients compared with single PCI treatment. Therefore, this combination therapy regimen is recommended for widespread clinical use. Meanwhile, in consideration of the limitations existing in this meta-analysis, high-quality and large-scale RCTs, with good experimental design and methodological quality, are needed to investigate the clinical effect and safety of CDDP for CHD in the future.

## 5. Conclusion

The results showed that CDDP combined with PCI treatment remarkably reduced the incidence of MACE in CHD patients. Meanwhile, this combination improved blood rheology, vascular endothelial function, and cardiac function, decreased blood lipid, and exhibited anti-inflammatory effects. However, our findings must be interpreted with care for the limitations existing in this meta-analysis. Other rigorous and large-scale RCTs are in need to confirm these results.

## Figures and Tables

**Table 1:** Characteristics of the included studies.

Study ID	T/C	Intervention	Control	CDDP dosage	Duration	Outcome measures
Wang et al. [19]	15/33	CDDP + control	PCI + conventional treatment	10 pills, t.i.d.	1 month	MACE; CF indexes
Qiu and Zhong [20]	32/30	CDDP + control	PCI + conventional treatment	10 pills, t.i.d.	3 months	CF indexes
Chen, 2008 [21]	30/30	CDDP + control	PCI + conventional treatment	10 pills, t.i.d.	3 months	BL and CF indexes
Xuan et al. [22]	50/50	CDDP + control	PCI + conventional treatment	10 pills, t.i.d.	3 weeks	VEF and CF indexes
Zhao et al. [23]	83/77	CDDP + control	PCI + conventional treatment	10 pills, t.i.d.	3 months	HR indexes
Li et al. [24]	252/248	CDDP + control	PCI + conventional treatment	10 pills, t.i.d.	1 month	MACE; CF indexes
Tian et al. [25]	44/40	CDDP + control	PCI + conventional treatment	10 pills, t.i.d.	3 months	MACE; BL indexes
Xia [26]	15/15	CDDP + control	PCI + conventional treatment	10 pills, t.i.d.	2 months	BL indexes
Tang and Zhang [27]	44/44	CDDP + control	PCI + conventional treatment	10 pills, t.i.d.	6 months	MACE; HR indexes
Yao et al. [28]	30/30	CDDP + control	PCI + conventional treatment	10 pills, t.i.d.	3 months	HR indexes
Li and Wang [29]	42/42	CDDP + control	PCI + conventional treatment	10 pills, t.i.d.	3 months	MACE; HR indexes
Fang et al. [30]	60/60	CDDP + control	PCI + conventional treatment	10 pills, t.i.d.	3 months	CF and inflammation indexes
Zhang [31]	76/84	CDDP + control	PCI + conventional treatment	10 pills, t.i.d.	3 months	MACE; CF indexes
Chen et al. [32]	60/60	CDDP + control	PCI + conventional treatment	10 pills, t.i.d.	1 month	BL and inflammation indexes
Li [33]	40/40	CDDP + control	PCI + conventional treatment	10 pills, t.i.d.	3 months	Inflammation indexes; ARs
Tian et al. [34]	79/79	CDDP + control	PCI + conventional treatment	10 pills, t.i.d.	1 month	MACE; VEF indexes; ARs
Wu and Xu [35]	97/97	CDDP + control	PCI + conventional treatment	10 pills, t.i.d.	1 month	VEF and inflammation indexes
Ji et al. [36]	67/69	CDDP + control	PCI + conventional treatment	10 pills, t.i.d.	1 week	MACE; CF indexes
Su [37]	75/75	CDDP + control	PCI + conventional treatment	10 pills, t.i.d.	2 months	CF indexes
Wang et al. [38]	90/90	CDDP + control	PCI + conventional treatment	10 pills, t.i.d.	2 months	CF and inflammation indexes

T, trial group; C, control group; CDDP, compound Danshen dripping pill; PCI, percutaneous coronary intervention; t.i.d., three times a day; MACE, major adverse cardiac events; HR, hemorheology; VEF, vascular endothelial function; BL, blood lipid; CF, cardiac function; conventional treatment referred to some Western medicines mainly including nitrates, *β*-blockers, calcium channel blockers, statins, and platelet inhibitors.

## Data Availability

The data used to support the findings of this study are included within the article.

## Abbreviations

CDDP: Compound Danshen dripping pill
CHD: Coronary heart disease
CI: Cardiac index
CVD: Cardiovascular disease
EAI: Erythrocyte aggregation index
ET: Endothelin
FIB: Fibrinogen
FMD: Flow mediated dilation
HCT: Hematocrit
HDL-C: High density lipoprotein cholesterol
Hs-CRP: High-sensitivity C-reactive protein
IL-6: Interleukin-6
IL-8: Interleukin-8
LDL-C: Low density lipoprotein cholesterol
LVEDD: Left ventricular end diastolic diameter
LVEF: Left ventricular ejection fraction
MACE: Major adverse cardiac events
NO: Nitric oxide
PCI: Percutaneous coronary intervention
PV: Plasma viscosity
RCTs: Randomized controlled trials
TC: Total cholesterol
TCM: Traditional Chinese medicine
TG: Triglyceride
TNF-*α*: Tumor necrosis factor-alpha
WBV: Whole blood viscosity.
